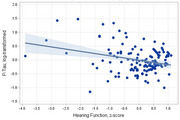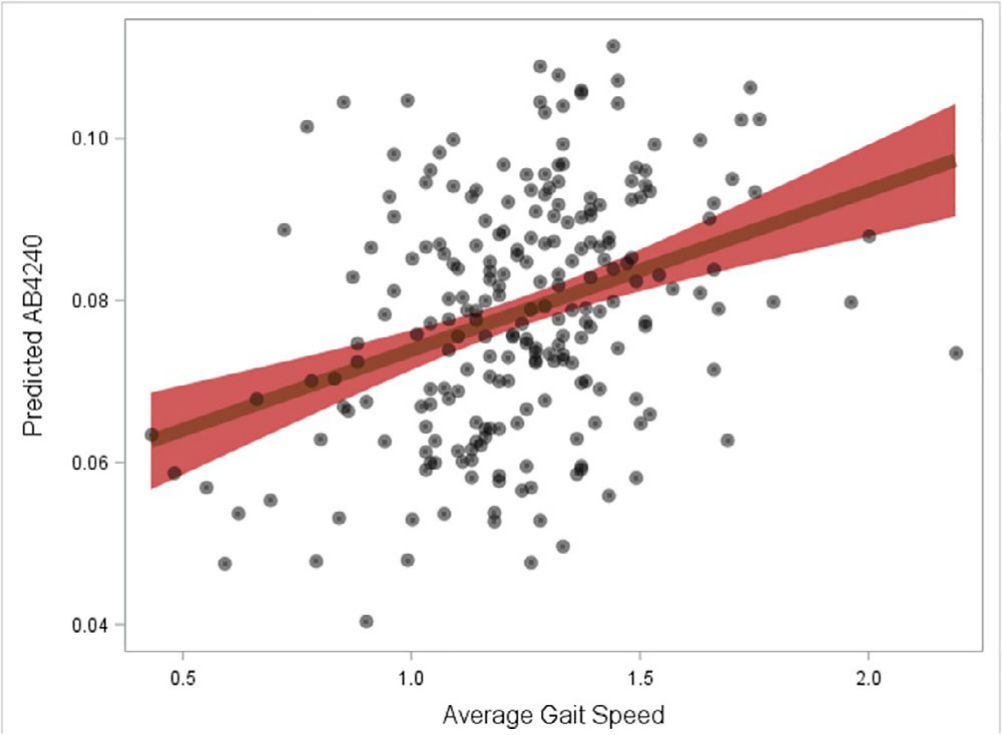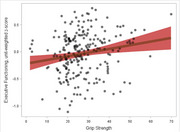# Sensory and Physical Function Associate with Cognition and Biomarkers for Alzheimer's Disease and Related Dementias Across the Lifespan

**DOI:** 10.1002/alz70856_098097

**Published:** 2025-12-24

**Authors:** Patrick J Smith, Kim G Johnson, Heidi L Roth, Guy G Potter, Sara Patillo, Weili J Lin, Allen J Song, Miles Berger, Richard J O'Brien, Andy Liu, Michael W Lutz, Sheng Luo, Andrea Bozoki, Kathleen A. Welsh‐Bohmer, Gwenn A Garden, Heather Whitson

**Affiliations:** ^1^ University of North Carolina at Chapel Hill, Chapel Hill, NC, USA; ^2^ Duke University, Durham, NC, USA; ^3^ UNC, Chapel Hill, NC, USA; ^4^ Duke University– Joseph and Kathleen Bryan Alzheimer's Disease Research Center, Durham, NC, USA; ^5^ Duke University Medical Center, Durham, NC, USA; ^6^ Duke/UNC Alzheimer's Disease Research Center, Duke University School of Medicine, Durham, NC, USA; ^7^ Duke University School of Medicine, Durham, NC, USA; ^8^ Duke Department of Neurology, Durham, NC, USA; ^9^ University of North Carolina, Chapel Hill, NC, USA

## Abstract

**Background:**

Worse sensory and physical function measures associate with higher risk of Alzheimer's Disease and Related Dementias (ADRD). To our knowledge, no studies have reported relationships between sensory and physical function, ADRD cerebrospinal fluid (CSF) biomarkers, and cognition in middle‐aged and younger adults. We examined these associations in the Duke‐UNC ADRC cohort.

**Method:**

We examined demographic and clinical correlates of ADRD biomarker profiles among participants enrolled in the Duke‐UNC ADRC (*n* = 243), among whom 67% underwent CSF biomarker assessments (*n* = 162). Visual function was assessed using canonical visual acuity assessments (Snellen, Jaeger, and Sloan Visual Acuity Tests and Dynamic Visual Function). Auditory function was assessed using the QuickSIN speech‐in‐noise test and Pure‐Tone audiometry. Physical function was assessed using grip strength and the Up‐and‐Go test. Cognitive function was assessed from the NACC Uniform Data Set. Factor analysis was used to aggregate individual cognitive subtests into two unit‐weighted domain scores: Executive, Language, and Visuospatial Function (ELVF) and Memory. Sensory function was combined by domain into unit‐weighted z‐scores. General linear models were used to examine the associations between sensory and physical function with cognitive function and ADRD biomarkers, controlling for age, education, gender, race, comorbidities, and APOE genotype.

**Result:**

Participants ranged from 28 to 80 years of age and tended to have normal cognitive performance. Better hearing (*p* <.001) and vision (*p* <.001) associated with better Memory performance, with similar associations for ELVF. Better hearing also associated with higher levels of Aβ42/40 (*B* = 0.26, *p* <.001) and lower levels of *p*‐tau181 (*B* = ‐0.28, *p* = .006; Figure 1). Higher gait speed associated with better Memory and ELVF (*p*s ≤ .040), as well as associating with higher Aβ42/40 (*B* = 0.21, *p* = .006; Figure 2) and lower NfL (*B* = ‐0.28, *p* = 002). Better grip strength associated with better ELVF (*B* = 0.20, *p* = .007; Figure 3).

**Conclusion:**

Sensory and physical function associate with both cognitive function and ADRD biomarkers among adults of widely varying ages, independent of background and clinical characteristics. Our analyses are likely underpowered to detect age‐varying effects and should therefore be replicated by other centers to ensure their robustness.